# Palytoxin and an *Ostreopsis* Toxin Extract Increase the Levels of mRNAs Encoding Inflammation-Related Proteins in Human Macrophages via p38 MAPK and NF-κB

**DOI:** 10.1371/journal.pone.0038139

**Published:** 2012-06-01

**Authors:** Rita Crinelli, Elisa Carloni, Elisa Giacomini, Antonella Penna, Sabrina Dominici, Cecilia Battocchi, Patrizia Ciminiello, Carmela Dell'Aversano, Ernesto Fattorusso, Martino Forino, Luciana Tartaglione, Mauro Magnani

**Affiliations:** 1 Section of Biochemistry and Molecular Biology, Department of Biomolecular Sciences, University of Urbino Carlo Bo, Urbino (PU), Italy; 2 Section of Environmental Biology, University of Urbino Carlo Bo, Urbino (PU), Italy; 3 Department of Chemistry of Natural Products, University of Napoli Federico II, Napoli, Italy; Instituto de Biofisica Carlos Chagas Filho, Universidade Federal do Rio de Janeiro, Brazil

## Abstract

**Background:**

Palytoxin and, likely, its analogues produced by the dinoflagellate genus *Ostreopsis*, represent a class of non-proteinaceous compounds displaying high toxicity in animals. Owing to the wide distribution and the poisonous effects of these toxins in humans, their chemistry and mechanism of action have generated a growing scientific interest. Depending on the exposure route, palytoxin and its *Ostreopsis* analogues may cause several adverse effects on human health, including acute inflammatory reactions which seem more typical of cutaneous and inhalation contact. These observations have led us to hypothesize that these toxins may activate pro-inflammatory signalling cascades.

**Methodology and Principal Findings:**

Here we demonstrate that palytoxin and a semi-purified *Ostreopsis* cf. *ovata* toxin extract obtained from a cultured strain isolated in the NW Adriatic Sea and containing a putative palytoxin and all the ovatoxins so far known – including the recently identified ovatoxin-f – significantly increase the levels of mRNAs encoding inflammation-related proteins in immune cells, i.e. monocyte-derived human macrophages, as assessed by Real-Time PCR analysis. Western immunoblot and electrophoretic mobility shift assays revealed that nuclear transcription factor -κB (NF-κB) is activated in cells exposed to toxins in coincidence with reduced levels of the inhibitory protein IκB-α. Moreover, Mitogen-Activated Protein Kinases (MAPK) were phosphorylated in response to palytoxin, as also reported by others, and to the *Ostreopsis* toxin extract, as shown here for the first time. By using specific chemical inhibitors, the involvement of NF-κB and p38 MAPK in the toxin-induced transcription and accumulation of Cycloxigenase-2, Tumor Necrosis Factor-α, and Interleukin-8 transcripts has been demonstrated.

**Conclusions and Significance:**

The identification of specific molecular targets of palytoxin and its *Ostreopsis* analogues, besides contributing to expand the still limited knowledge of the intracellular signalling cascades affected by these toxins, may have important implications in setting up focused pharmacological interventions, replacing currently used symptomatic treatments.

## Introduction

Palytoxin (PLTX) is a potent non-protein marine toxin isolated in 1971 from *Palythoa*, a soft coral of the Pacific Ocean [1]–??[3]. Since then, PLTX and a number of palytoxin analogues have been extracted from many other marine organisms, including those belonging to the dinoflagellate genus *Ostreopsis* [4]. *Ostreopsis* species are important components of the tropical and subtropical reef environments, however, recently they have spread to temperate waters. In the last few years, massive blooms of *Ostreopsis* cf. *ovata* (*O*. cf. *ovata*), represented by the genotype of the Atlantic/Mediteranean clade [5], have been observed along the Mediterranean coasts [6]–??[8]. High resolution liquid chromatography-mass spectrometry (HR LC-MS) studies disclosed the presence, both in field and cultured *O.* cf. *ovata* cells, of putative PLTX [9] and six new palytoxin congeners, named ovatoxins (OVTX), namely OVTX-a [10], OVTX-b, -c, -d + -e and -f [11], [12]. Palytoxin and its analogues may enter the food chain and accumulate mainly in fishes and crabs, causing severe human intoxication and death following ingestion of contaminated products [13], [14]. Furthermore, toxic effects in individuals exposed via inhalation or skin contact to marine aerosol in coincidence with *Ostreopsis* blooms, have been reported [15], [16]. Thus, the formerly unsuspected broad distribution of the benthic dinoflagellate *Ostreopsis* spp. has recently posed a problem of risk assessment for human health [17], [18].

At the cellular level, the Na^+^/K^+^–ATPase is the primary molecular target of PLTX. To this regard, the ability of palytoxin to bind the Na^+^/K^+^–ATPase and convert it into a non-selective ion channel, has been widely demonstrated in various experimental systems [19], [20]. The transformation of the Na^+^/K^+^–ATPase into a cation channel is associated with a series of secondary effects, including disruption of the ion equilibrium, increased Na^+^ permeability, membrane depolarization and consequent Ca^2+^ influx that may lead to multiple events regulated by Ca^2+^-dependent pathways [21]. Depending on the cell type and toxin dose, filamentous actin (F-actin) disassembly, cell rounding and swelling, and cell death, have been described [22]–??[24].

Palytoxin has also been demonstrated to act as a non-TPA (12-*O*-tetradecanoylphorbol-13 acetate)-type skin tumor promoter, being able to modulate key signal transduction pathways involved in carcinogenesis [25], [26]. In particular, it has been shown that PLTX stimulates prostaglandin production from arachidonic acid [27] and activates MAPKs (Mitogen-Activated Protein Kinases), including extracellular signal-regulated kinase (ERK), c-Jun N-terminal kinase (JNK) and p38 MAPK [28]. MAPKs are a family of serine/threonine kinases that mediate intracellular signaling associated with a variety of cellular activities such as cell proliferation, differentiation, survival, death, and transformation [29]. Mitogenic agents typically activate ERK, while p38 and JNK signaling pathways are activated by inflammatory cytokines or in response to cellular stresses such as genotoxic, osmotic, hypoxic or oxidative stress. Once activated, MAPKs can phosphorylate various protein substrates, including transcription factors, and thereby modulate gene expression. Unfortunately, as far as palytoxin is concerned, the knowledge of the biochemical pathways by which MAPKs activation is transmitted to downstream effectors and nuclear targets and translated into biological outcomes is still limited. Most of the studies concerning this aspect have been performed by Wattemberg's research group which is interested in PLTX as tool to probe the role of different types of signalling mechanisms in carcinogenesis [28]. On the other hand, a deeper understanding of the mechanisms of action and of the signal transduction cascades affected by PLTX and its derivatives would help to better interpret the effects observed in subjects coming into contact with these biotoxins and to set up rational pharmacological interventions to limit or avoid systemic adverse reactions. Interestingly, a febrile-respiratory syndrome has been observed in individuals exposed to *O.* cf. *ovata* bloom aerosols; the symptoms included rhinorrhea, cough, fever and asthma-like illness (reviewed in [18]). In addition, PLTX application to the skin caused a severe irritative reaction, involving inflammation, edema and necrosis in animals [30]. Cases of dermal toxicity (edema erythema, urticarial rush, pruritus) have also been documented in humans exposed to marine water containing *O.* cf. *ovat*a cells or in patients who have handled zoanthid corals [31]. On the whole, these observations strongly suggest that PLTX and its congener toxins may actively engage and modulate pro-inflammatory signaling pathways, leading to production of inflammatory mediators in immune cells.

In this study, we provide the very first evidence that palytoxin is able to increase the levels of mRNAs encoding inflammation-related proteins in primary human macrophages through activation of p38 MAPK and transcription factor –κB (NF-κB). Macrophages play a critical role in the initiation, maintenance, and resolution of inflammation [32]. By synthesizing and secreting a wide array of cytokines (including interleukins-1, -6, and tumor necrosis factor), chemokines (including interleukin-8), and arachidonic metabolites, macrophages initiate inflammatory responses and recruit activated neutrophils to the site of inflammation. NF-κB is a well known transcription factor which plays a crucial role in the transcriptional regulation of genes involved in controlling cell survival and death, inflammation, and stress responses [33]. In particular, the NF-κB pathway is considered a prototypical pro-inflammatory signaling pathway, largely because of the role of NF-κB in the expression of genes such as those encoding for cytokines, chemokines and adhesion molecules [34].

Interestingly, the same effects produced at the molecular level by PLTX were also observed when a semi-purified toxin extract, obtained from cultured O. cf. *ovata* cells, was tested, suggesting that toxins contained in the extract may have a biological activity similar to that displayed by PLTX. Quali-quantitative composition of ovatoxins depends on the *O.* cf. *ovata* strain: in most cases OVTX-a, -b, -c, and –d + -e are synthesized by the alga [11], [35]–??[37] and only very recently a strain producing all these ovatoxins together with OVTX-f has been found [12]. This *O.* cf. *ovata* strain, that is quite unique in that synthesizes all the ovatoxins so far known, was used in this study. To the best of our knowledge, this is the first attempt to gain insights into the mechanism of action of *Ostreopsis* toxins.

## Results

### Toxin profile of the *O.* cf. *ovata* extract

**Figure 1:**
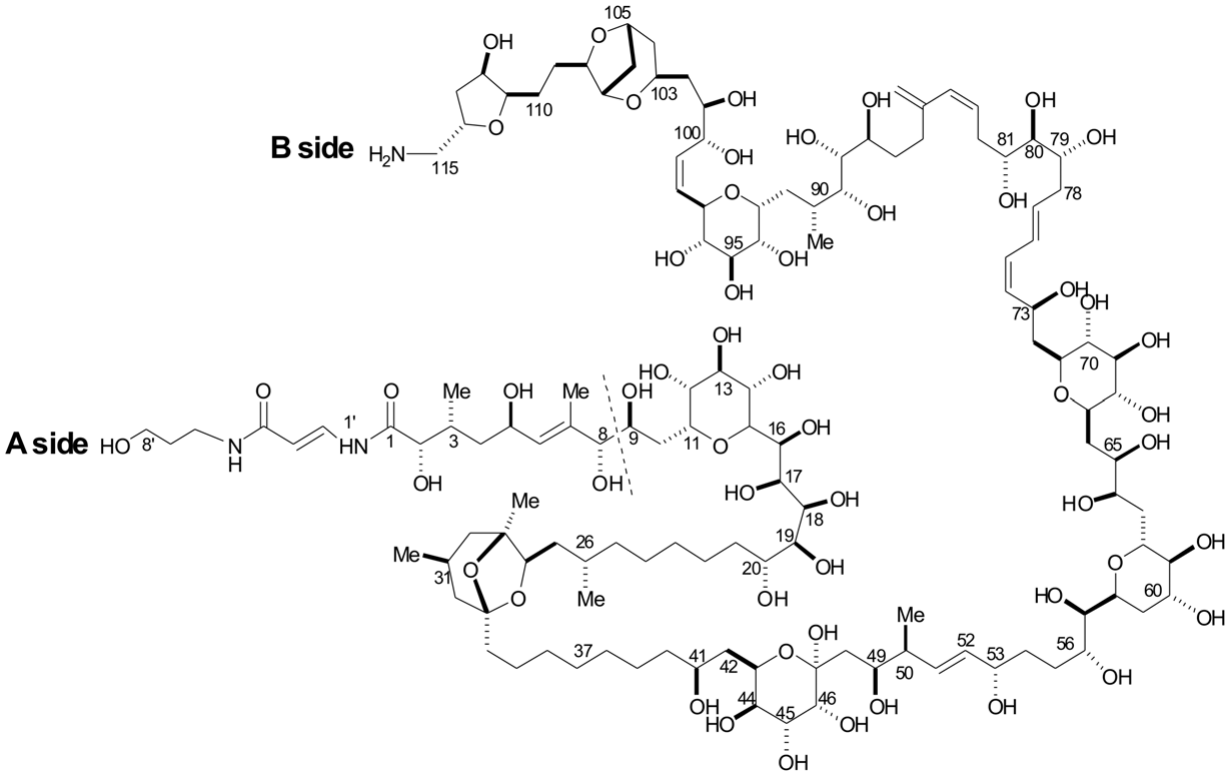
Structure of palytoxin. Cleavage between C-8 and C-9 occurs in HR LC-MS and MS^2^ experiments and divides the molecule in two moieties, A-side and B-side.

An extract obtained from *O.* cf. *ovata* CBA2-122, a strain isolated in the north-western Adriatic Sea Ancona- Italy, was subjected to a single clean-up step and used in this study. Further purification was avoided in order to recover enough toxins to perform *in vitro* studies. The semi-purified extract contained a putative PLTX and all the ovatoxins so far known, namely OVTX-a, -b, -c, -d + -e and -f [11], [12]. Ovatoxins have recently been identified as palytoxin-like compounds based on a comparison of their HR LC-MS data with those of palytoxin, namely i) retention times, ii) molecular formulae from cross-checking of their respective [M+H]^+^, [M+2H-H_2_O]^2+^, and [M+H+Ca]^3+^ ions, and iii) elemental composition of fragment ions from the favored C-8 and C-9 cleavage dividing palytoxin-like molecules in two partial structures A and B, respectively (Table 1 and Figure 1). Note that herein we refer both in name and elemental composition to ovatoxin-b, -c, -d + -e previously described by Ciminiello et al. [11]. According to the reported HR LC-MS and MS^n^ data [10], [11] i) OVTX-a presents the same A-moiety as PLTX and 2 oxygen atoms fewer (potentially 2 hydroxyl groups) in the B-moiety; ii) OVTX-b presents C_2_H_4_O (potentially a hydroxyl and two methylene groups) more than OVTX-a in the A moiety whereas structure B is identical, at least in the elemental composition; iii) OVTX-c presents C_2_H_4_O_2_ more than OVTX-a. Compared to ovatoxin-a, it presents additional C_2_H_4_O atoms (potentially a hydroxyl and two methylene groups) in the A moiety and an extra oxygen atom (potentially a hydroxyl group) in the B moiety; iv) OVTX-d and -e are isobaric compounds that present one oxygen atom more than OVTX-a. OVTX-d presents the same A moiety as OVTX-a and one additional oxygen atom (potentially a hydroxyl group) in the B moiety, while OVTX-e contains one more oxygen atom (potentially a hydroxyl group) in the A moiety and the same B moiety as OVTX-a; v) ovatoxin-f presents the same A-moiety as OVTX-a and C_2_H_4_ more than ovatoxin-a (potentially two methylene or methyl groups) in the B-moiety. This last toxin has very recently been identified in the *O.* cf. *ovata* strain used in this study and subjected to an in-depth HR LC-MS^n^ study which allowed the restriction of the elemental composition difference between OVTX-a and OVTX-f to the region close to the B-side terminal [12].

**Table 1:** Most abundant ions of palytoxin and ovatoxins used in quantitative HR LC-MS analyses.

TOXIN	Principal ions (*m*/*z*)		Elemental formulae		
name	[M+2H-H_2_O]^2+^	[M+H+Ca]^3+^	M	A side	B side
palytoxin	1331.2417	906.4851	C_129_H_223_N_3_O_54_	C_16_H_28_N_2_O_6_	C_113_H_195_NO_48_
ovatoxin-a	1315.2480	895.8255	C_129_H_223_N_3_O_52_	C_16_H_28_N_2_O_6_	C_113_H_195_NO_46_
ovatoxin-b	1337.2595	910.4976	C_131_H_227_N_3_O_53_	C_18_H_32_N_2_O_7_	C_113_H_195_NO_46_
ovatoxin-c	1345.2566	915.8286	C_131_H_227_N_3_O_54_	C_18_H_32_N_2_O_7_	C_113_H_195_NO_47_
ovatoxin-d	1323.2439	901.1533	C_129_H_223_N_3_O_53_	C_16_H_28_N_2_O_6_	C_113_H_195_NO_47_
ovatoxin-e	1323.2439	901.1533	C_129_H_223_N_3_O_53_	C_16_H_28_N_2_O_7_	C_113_H_195_NO_46_
ovatoxin-f	1329.2606	905.1616	C_131_H_227_N_3_O_52_	C_16_H_28_N_2_O_6_	C_115_H_199_NO_46_

Molecular formulae (M) of each compound and elemental composition of their relevant A- and B-side fragments deriving from cleavage between C-8 and C-9 (see Figure 1), as deduced by HR LC-MS and MS^2^ experiments.

Toxins contained in the semi-purified extract, (here referred to as OSTRTX – *Ostreopsis* Toxins), were quantified as reported in the experimental section. OSTRTX concentration in the Ancona *O.* cf. *ovata* semi-purified extract was calculated to be 3.59 µg/ml. Ovatoxin-f was the principal component of the toxin profile (46.6%) followed by ovatoxin-a (21.7%), ovatoxin-b (19.5%), ovatoxin-c (2.6%), ovatoxin-d + -e (8.4%) and putative palytoxin (1.2%). Toxin profile is quite unique as it comprises all the ovatoxins so far identified.

### Increased levels of Cycloxygenase-2, Tumor Necrosis Factor-α and Interleukin-8 mRNAs in monocyte-derived human macrophages upon exposure to palytoxin and to the OSTRTX extract

To test whether palytoxin and palytoxin-like compounds might trigger a pro-inflammatory response, a commercially available PLTX standard and the semi-purified toxin extract obtained from *O.* cf. *ovata* CBA2-122 (both quantified by HR LC-MS) were administered to monocyte-derived human macrophages and the mRNA level of threedifferent inflammation-related genes – Cycloxigenase-2 (COX-2), Tumor Necrosis Factor-α (TNF-α) and Interleukin-8 (IL-8) – was monitored by Real-Time PCR analysis.

Macrophages were selected as the experimental system since they play a critical role in the initiation, maintenance, and resolution of inflammation. Furthermore, we chose to use primary cells rather than cell lines because monocyte-derived macrophages, similarly to tissue macrophages, are terminally differentiated and do not proliferate; thus, they are more relevant as a macrophage model than immortalized cell lines. Indeed, in literature, considerable differences in response to stimuli between primary cells and different cell lines have been well documented [38].

**Figure 2:**
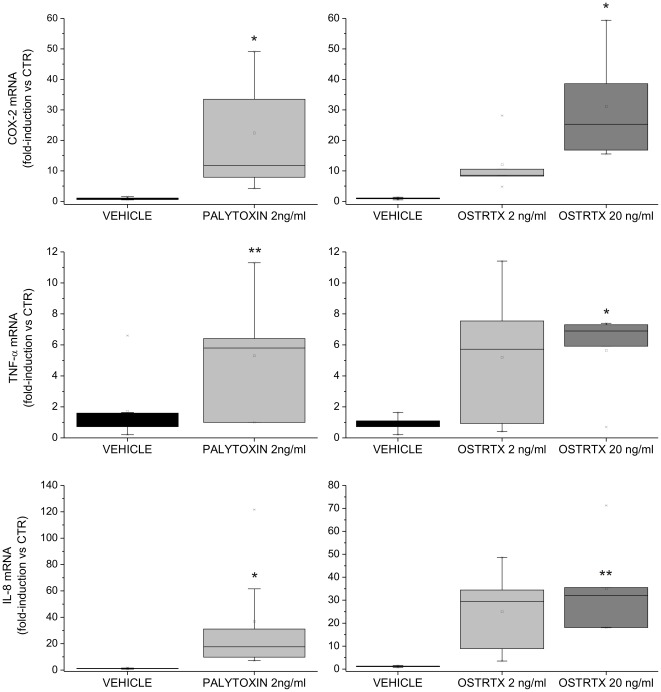
COX-2, IL-8 and TNF-α mRNA levels in human macrophages exposed to PLTX and to the OSTRTX extract. Evaluation of COX-2, IL-8 and TNF-α mRNA levels by quantitative Real-Time PCR assay. Total RNA, extracted from primary human macrophages exposed 4 h to PLTX (2 ng/ml), to the *O*. cf. *ovata* toxin extract (final OSTRTX concentration 2 ng/ml and 20 ng/ml) or the vehicle (i.e. 0.05% MetOH), was amplified with gene-specific primers. Expression data, normalized to the housekeeping B2M gene, were analyzed by the 2^−ΔΔCT^ method and referred to the value obtained in untreated cells (CTR). The boxplots show the results of 8 and 5 independent experiments performed with PLTX and the OSTRTX extract, respectively. Asterisks indicate statistical significance versus cells receiving the vehicle alone (*p<0.05; **p<0.01).

As shown in Figure 2, messenger RNA levels of all the selected target genes were up-regulated after 4 h from PLTX administration. At shorter time points (i.e. 1 h and 2 h) no differences were detected between control and toxin-treated cells (data not shown). It is worth noting that there was a certain heterogeneity in the amount of transcripts produced in response to toxins between macrophages derived from different cell donors. This donor-to-donor variation probably reflects the diversity in the immune response between individuals. Nevertheless, a statistically significant increase in COX-2, TNF-α and IL-8 mRNA cellular content over the level present in vehicle-treated cells, was found in macrophages exposed to 2 ng/ml of palytoxin, corresponding to 0.75 nM toxin. Similar results were obtained when the semi-purifiied *O.* cf. *ovata* toxin extract was tested, although target gene mRNA levels were significantly increased as compared to the control only if the OSTRTX dose was raised from 2 ng/ml to 20 ng/ml (Figure 2). Indeed, incubation of cells with 2 ng/ml OSTRTX did not result in a statistically significant up-regulation of transcript cellular content (Figure 2). Importantly, the vehicle alone (i.e. 0.05% Methanol) had no effects, thus demonstrating specificity of toxin action (Figure 2). Furthermore, since typical macrophage activators are bacterial endotoxins, which represent the most common contaminants of research laboratory materials and reagents, PLTX stock solutions and the OSTRTX extract were tested with a commercial Limulus assay. This kind of test revealed that toxin preparations were endotoxin-free, thus allowing the exclusion of the possibility that the observed effects were due to bacterial contaminants rather than to the marine biotoxins themselves.

Since mRNA accumulation is expected to result in protein production, the levels of COX-2, TNF-α and IL-8 proteins were monitored by western immunoblotting and immunoprecipitation of the cytokines from the culture medium, using specific antibodies. Unfortunately, attempts to demonstrate COX-2, TNF-α and IL-8 protein expression were unsuccessful. Indeed, no proteins were detected in toxin-treated cells, neither at 4 h of incubation, in coincidence of mRNA appearance, nor at later incubation times (i.e. 8 h, 16 h and 24 h), when they are expected to accumulate within cells or in the culture medium in sufficient amounts to be revealed by conventional methods (data not shown). By contrast, COX-2, TNF-α and IL-8 proteins were always detectable in lipopolysaccharide-stimulated macrophages, used as a positive control, thus demonstrating the efficacy of the experimental approaches employed (data not shown).

**Figure 3:**
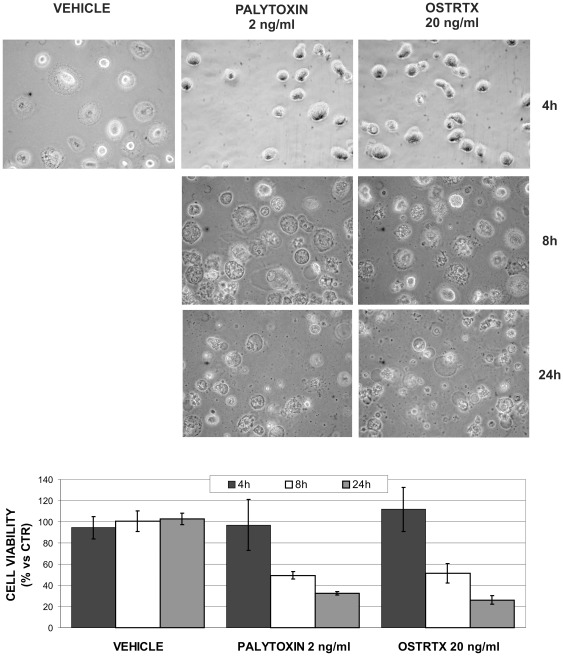
Macrophage morphology and viability after exposure to PLTX and to the OSTRTX extract. Optical images of macrophages incubated 4 h, 8 h and 24 h with PLTX (2 ng/ml) and the *O*. cf. *ovata* toxin extract (final OSTRTX concentration 20 ng/ml) versus cells receiving the vehicle alone (i.e. 0.05% MetOH). Pictures were taken with a magnification X 40 lens. Cell viability was measured at 4 h, 8 h and 24 h incubation with the toxins by the MTS assay. All the values are referred to cells left untreated (CTR). The graph shows the results of at least 3 independent experiments (± S.E.M.).

However, it should be highlighted that while at 4 h incubation cells underwent rounding, but were still metabolically active, as assessed by MTS assay (Figure 3), 50% of the cell population died within 8 h of exposure to both PLTX and OSTRTX and conspicuous vacuoles appeared in the cytoplasm of residual adherent cells (Figure 3). At 24 h, about 25% of the macrophages were still alive, but most of them contained a big unique vacuole, occupying almost the entire cell volume (Figure 3). This phenotype is reminiscent of autophagic processes which usually precede or accompany cell death [39], although further experiments will be necessary to determine the exact mechanism.

It has been demonstrated that PLTX can act as a potent translational inhibitor, being able to completely inhibit protein synthesis in Rat-1 cells at a dose of 10 pM [40]. Thus, in light of the finding that toxic effects occur very rapidly after exposure to the toxins and that cells may become unable to efficiently translate mRNAs into proteins, we concluded that the toxin doses used in our study may not allow to demonstrate that mRNAs encoding for COX-2, TNF-α and IL-8 are indeed translated into their respective protein products.

### PLTX and the OSTRTX extract lead to reduced IκB-α protein levels and increased IκB-α mRNA, suggesting that toxins may induce activation of NF-κB

**Figure 4:**
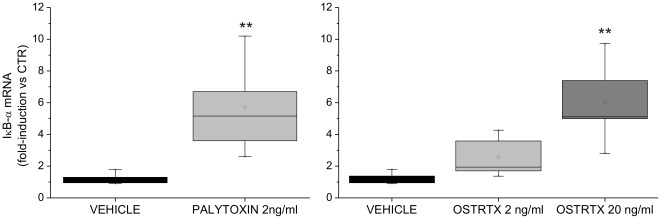
IκB-α mRNA levels in human macrophages exposed to PLTX and to the OSTRTX extract. Evaluation of IκB-α mRNA levels by quantitative Real-Time PCR assay. Total RNA, extracted from primary human macrophages exposed 4 h to PLTX, to the *O*. cf. *ovata* toxin extract (final OSTRTX concentration 2 ng/ml and 20 ng/ml) or to the vehicle (i.e. 0.05% MetOH), was amplified with IκB-α gene-specific primers. Expression data, normalized to the housekeeping B2M gene, were analysed by the 2^−ΔΔCT^ method and referred to the value obtained in untreated cells (CTR). The boxplots show the results of 8 and 5 independent experiments performed with PLTX and the OSTRTX extract, respectively. Asterisks indicate statistical significance versus cells receiving the vehicle alone (*p<0.05; **p<0.01).

**Figure 5:**
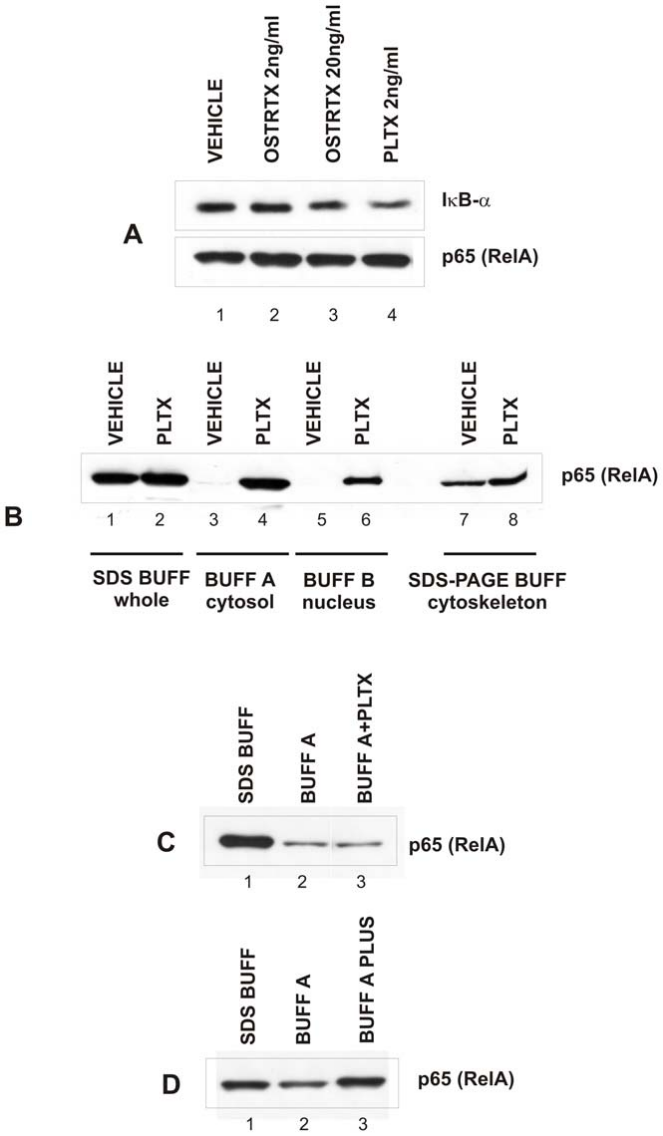
IκBα and p65 protein levels in cells treated with the vehicle, the OSTRTX extract and PLTX. (**A**) IκBα protein levels were determined in whole cell lysates obtained from macrophages incubated 4 h with the vehicle (lane 1), the OSTRTX extract (lanes 2–3) or PLTX (lane 4). Protein extracts (15 µg) were resolved by SDS-PAGE on 8% gel and then submitted to Western immunoblotting. Blots were probed with an anti-IκBα (upper panel) and an anti-p65 (RelA) antibody to check for protein loading (lower panel). (**B**) intracellular content and subcellular distribution of p65 (RelA) in PLTX- versus vehicle-treated cells was assessed by immunoblotting analysis of whole (15 µg, lanes 1–2), cytosolic (15 µg, lanes 3–4) and nuclear (10 µg, lane 5–6) extracts. Whole cell extracts were obtained by lysing cells in SDS buffer. In parallel, cells were sub-fractionated by extraction in Buffer A (BUFF A, cytosolic proteins) followed by Buffer B (nuclear proteins, BUFF B), the residual material, containing insoluble proteins, including those associated with cytoskeletal structures, was solubilized in SDS-PAGE sample buffer and run in parallel (lanes 7–8). (**C**) Approaches to inhibit p65 *in vitro* degradation during extraction in native conditions. Cell pellets, containing an equivalent number of macrophages, were lysed in SDS buffer (SDS BUFF, lane 1), Buffer A (BUFF A, lane 2) or Buffer A supplemented with palytoxin (BUFF A+PLTX, lane 3). An equal volume of SDS sample buffer was added to all tubes and comparable volumes of the resulting protein extracts were resolved by electrophoresis and immunoblotted with an anti p65 antibody.(**D**) cell pellets as in C were lysed in SDS buffer (lane 1), Buffer A (lane 2) and Buffer A further supplemented with lysososmal protease inhibitors (BUFF A PLUS, lane 3) and submitted to SDS-PAGE and immunoblotting with an anti p65 antibody.

NF-κB is a transcription factor which controls the expression of numerous genes involved in immune and inflammatory responses. The classical NF-κB complex is a heterodimer composed of the p65/RelA and p50 subunits. NF-κB is retained in the cytoplasm of most cells due to association with inhibitory proteins, called IκBs, of which the most common is IκB-α. Upon cell stimulation, IκB-α is phosphorylated, ubiquitinated and degraded within the cytosol by the 26S proteasome complex, thus NF-κB subunits are released and translocate to the nucleus where they turn on target gene expression [41]. One of the first genes to be transcribed is that encoding IκB-α itself [42]. Newly synthesized free IκB-α binds to nuclear NF-κB leading to export of the complex into the cytoplasm, thus allowing the establishment of a negative feedback loop which controls NF-κB activation [43], [44]. In order to assess the possibility that NF-κB may be activated by PLTX and the OSTRTX extract, we monitored the levels of IκB-α mRNA in cells receiving 4 h treatment with PLTX (2 ng/ml) or with the *O.* cf. *ovata* toxin extract to a final OSTRTX concentration of 2 ng/ml and 20 ng/ml. Since IκB-α gene promoter is indeed strictly dependent on functional NF-κB, IκB-α mRNA induction represents a bona fide marker of NF-κB activation. Real-Time PCR analysis revealed that IκB-α mRNA levels were significantly increased over the basal level upon stimulation with 2 ng/ml PLTX or 20 ng/ml OSTRTX, thus suggesting that NF-κB is activated by both toxin preparations (Figure 4). Although this evidence demonstrates that, at 4 h incubation, IκB-α re-synthesis had already occurred, IκB-α protein levels were still lower in PLTX- and OSTRTX-treated cells than in cells receiving the vehicle or ineffective doses of OSTRTX (i.e. 2 ng/ml), as assessed by western immunoblot of whole cell lysates with a specific antibody (Figure 5A, upper panel, compare lanes 3 and 4 with 1 and 2). As loading control, the blot was re-probed with an anti p65 antibody since, upon NF-κB activation, p65 redistributes within intracellular compartments while the whole content does not change. Accordingly, no differences in total p65 protein levels were observed (Figure 5A, lower panel). Since protein extracts of Figure 5A were obtained by lysing the cells in SDS (Sodium Dodecyl Sulphate) buffer which allows to immediately boil the sample and denature proteins, we excluded that reduced levels of IκB-α could be the consequence of *in vitro* degradation processes. On the whole, this demonstrates that toxins are presumably able to activate NF-κB by reducing the levels of its inhibitory protein IκB-α. Despite increased mRNA levels, the cellular content of IκB-α protein remained low, probably for the same reason why we could not detect COX-2, TNF-α and IL-8 proteins.

### NF-κB p65 subunit is degraded *in vitro* by lysosomal proteases the activity of which is inhibited *in vivo* by PLTX and the OSTRTX extract

Since IκBα degradation leads to NF-κB nuclear translocation, PLTX-, OSTRTX- or vehicle-treated cells were sub-fractionated in order to obtain nuclear extracts to be tested in EMSA (Electrophoretic Mobility Shift Assay). This type of assay, also known as bandshift, is a rapid and sensitive method to detect and quantify transcription factors in crude extracts, based on their ability to bind DNA target sequences *in vitro*. Before setting up bandshift assays, cytoplasmic-nuclear fractions were preliminary subjected to western immunoblot analysis, using an antibody specific for p65/RelA. Surprisingly, while p65 was detected in whole lysates of vehicle-treated cells (Figure 5B, lane 1), obtained by disrupting the cells directly in denaturing SDS buffer as in Figure 5A, the immunoreactive band was almost undetectable in cytosolic (Figure 5B, lane 3) and nuclear (Figure 5B, lane 5) fractions, deriving from an extraction protocol in native conditions. By contrast, p65 was clearly present in both compartments of PLTX- (Figure 5B, lanes 4 and 6) and OSTRTX-treated cells (data not shown) and the amount detected was consistent with the levels determined in whole cell extracts (Figure 5B, lane 2). p65/RelA has been found associated with the cytoskeleton [45] and palytoxin is known to alter cytoskeletal dynamics [46]. Therefore, we have hypothesized that, in vehicle-treated cells, p65 precipitated in the insoluble fraction together with the cytoskeletal structures. By contrast p65 was released in the soluble fraction in cells receiving PLTX, due to the ability of the toxin to depolarize actin fibers. To this end, the pellet obtained after nuclear protein extraction, which should contain the cytoskeleton and which is usually discarded, was solubilized in SDS-PAGE (Polyacrilamide Gel Electrophoresis) sample buffer and submitted to western immunoblotting analysis. As shown in figure 5B, again p65 content was lower in cells receiving the vehicle than in PLTX-treated macrophages (compare lane 7 with 8). Based on these observations, we concluded that p65 was not differently distributed within the cell, but rather its levels were lower in control than in toxin-treated cells. Indeed, during cytosolic extraction in native conditions, active lysosomal proteases are released from lysosomes and because they are not denatured, they are free to degrade cytosolic and nuclear proteins. Interestingly, the activity of these proteases was not neutralized by the commercial cocktail of protease inhibitors used, but was apparently inhibited (directly or indirectly) by PLTX and OSTRTX treatment. Thus, we first checked whether PLTX addition to extraction buffers, in particular that used to break cells and release the cytosolic content (Buffer A), was sufficient to block *in vitro* protein degradation. To this purpose, a suspension of untreated cells was divided into three tubes, and the cell pellets were extracted in an equal volume of SDS buffer, Buffer A and Buffer A supplemented with 8 ng/ml PLTX (a 4-fold excess with respect to the amount added in the culture medium), respectively. The sample in SDS buffer was immediately boiled and diluted with an equal volume of buffer A, while the other two were incubated on ice for 30 min. At the end of the incubation time, an equal volume of SDS sample buffer 2× was added to all tubes and the samples boiled in order to obtain comparable protein patterns. Western blotting analysis of the samples revealed that no differences in p65 protein levels were found between macrophages extracted in Buffer A (Figure 5C, lane 2) and Buffer A containing PLTX (Figure 5C, lane 3). In both cases the amount of p65 was far lower than that detected in cells lysed directly in SDS buffer (Figure 5C, compares lanes 2 and 3 with lane 1). From this experiment, we concluded that PLTX is probably able to exert an inhibitory activity on lysosomal proteases *in vivo* by an indirect mechanism. This aspect, although interesting and noteworthy, was no further investigated because it was beyond the scope of the present study. By contrast, to continue our analyses, it was necessary to inhibit p65 degradation during extractions in native conditions in order to quantitatively compare nuclear extracts obtained from vehicle-treated and PLTX/OSTRTX-treated cells when submitted to EMSA.

It is well known that endosomes and lysosomes harbor serine, cysteine and aspartic acid proteases. Thus, to block their activity, all the extraction buffers were further supplemented with specific chemical inhibitors such as leupeptin, pestatin, AEBSF [4-(2-aminoethyl)-benzenesulfonyl fluoride] and MG-132. To test the efficacy of the new protease inhibitor cocktail, we repeated the experiment above by lysing the cells in SDS buffer, Buffer A and Buffer A PLUS (i.e. Buffer A containing the commercial and the new proteasome inhibitor cocktail). This buffer formulation was effective in preventing p65 degradation as shown in Figure 5, panel D, where it is possible to note that levels of p65 in cells lysed in Buffer A PLUS (line 3) were significantly higher than those obtained in Buffer A (line 2) and very similar to those detected by lysing cells in SDS buffer (line 1). In this case as well, comparisons between protein content were made possible by adding an equal volume of Buffer A or SDS buffer to SDS or Buffer A/A PLUS lysates, respectively. Based on the above results, EMSA experiments were subsequently performed using nuclear extracts obtained by supplementing all the extraction buffers with the new inhibitor cocktail, as reported under “Materials and Methods”.

### PLTX and the OSTRTX extract induce NF-κB nuclear translocation

**Figure 6:**
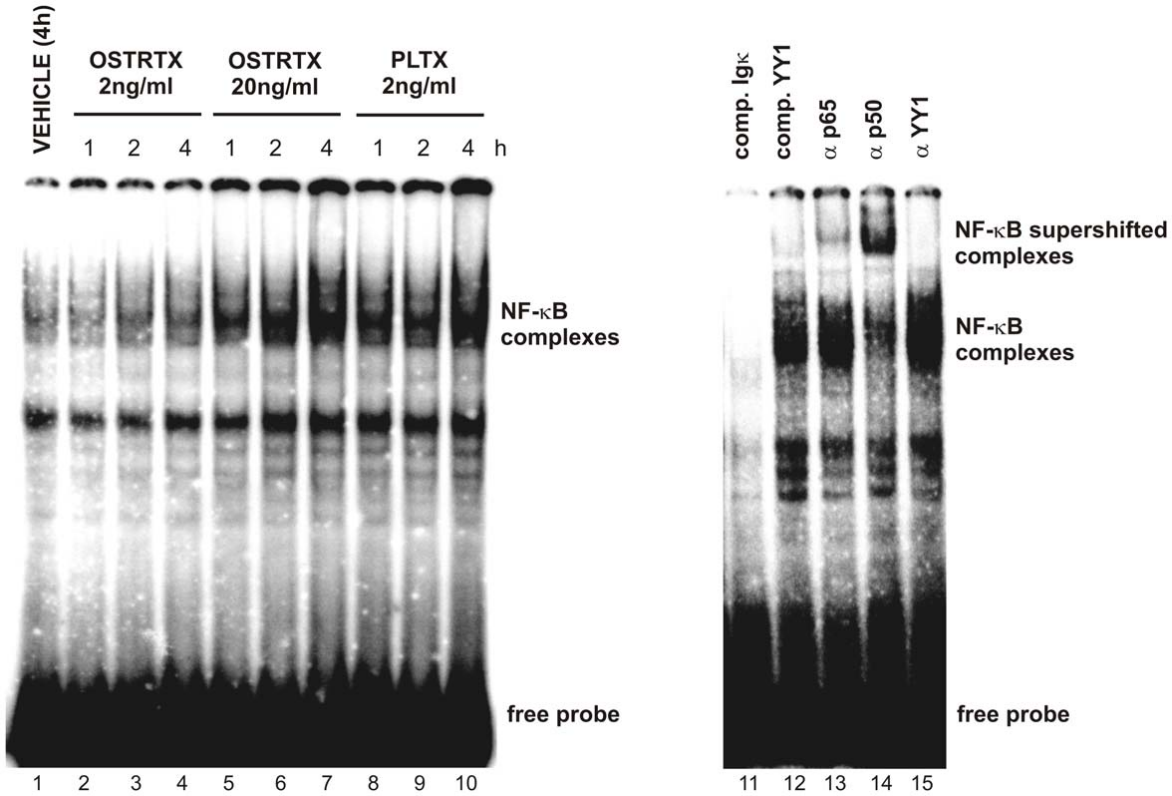
NF-κB nuclear translocation in toxin-treated cells. Nuclear extracts were obtained from macrophages incubated with the *O*. cf. *ovata* toxin extract (final OSTRTX concentration 2 ng/ml and 20 ng/ml) (lanes 2–7) or PLTX (2 ng/ml) (8–10) for different lengths of time (i.e. 1 h, 2 h and 4 h). Cells exposed to the vehicle 4 h were used as control (lane 1). Nuclear extracts (5 µg) were submitted to EMSA using a ^32^P-labeled ODN, containing the NF-κB consensus sequence (Igκ), as probe. Protein-DNA complexes were separated on 5% PAGE and then detected in a GS-250 Molecular Imager. Specificity of binding was assessed by competition experiments where nuclear extracts, from PLTX-treated cells, were pre-incubated with a 50-fold excess of cold Igκ probe (lane 11) or an ODN containing the consensus sequence of the unrelated factor YY1 (lane 12). For supershift assay nuclear extracts were pre-incubated with antibodies against NF-κB p65 and p50 subunits (lanes 13, 14), while an antibody against YY1 was used as control (lane 15).

Once established the conditions to prevent *in vitro* degradation processes, nuclear extracts were prepared from macrophages treated with the *O.* cf. *ovata* toxin extract at effective (20 ng/ml) and ineffective (2 ng/ml) OSTRTX doses, as previously determined, and with palytoxin (2 ng/ml) for different lengths of time up to 4 h. As control, cells were exposed 4 h to the vehicle. Nuclear extracts were incubated with a double-stranded oligonucleotides (Igκ)- ^32^P-labelled containing the NF–κB consensus sequence, and then submitted to native PAGE (Figure 6). NF-κB/DNA complexes were detected by exposing the gel in a Molecular Imager. The resulting image clearly shows that NF-κB translocates into the nucleus of both OSTRTX- and PLTX-treated cells as soon as 1 h after toxin addition to the culture medium, as demonstrated by an increase in the signal of the retarded band over the basal level (Figure 6, lanes 5–7 and 8–10). The highest signal was obtained at 4 h (lanes 7 and 10). NF-κB nuclear translocation was only slightly detectable in the nucleus of the cells receiving the dose of OSTRTX which did not result in a significant increase in the mRNA levels of inflammation-related genes (Figure 6, lanes 2–4). Specificity of the assay was demonstrated by disappearance of the signal when a 50-fold excess of unlabelled Igκ, but not of an unrelated ODN (YY1) was included in the reaction mixture (Figure 6, lanes 11 and 12), before adding the labeled probe. Supershift analysis revealed that toxin-induced NF-κB complexes contained p65 and p50 subunits, as demonstrated by up-shift of the signal following addition of specific antibodies (Figure 6, lanes 13, 14). No shift was observed with an antibody against an unrelated factor (YY1) (Figure 6, lane 15).

### NF-κB and p38 MAPK signaling pathways are activated by PLTX and the OSTRTX extract to increase the mRNA levels of inflammation-related genes

To verify whether NF-κB activation was part of the molecular mechanism(s) mediating PLTX- and OSTRTX-induced COX-2, TNF-α and IL-8 mRNA expression and accumulation, cells were pre-treated with andrographolide, a molecule able to interfere *in vivo* with the binding of NF-κB to endogenous DNA consensus sequences, and then incubated 4 h with the toxins.

**Figure 7:**
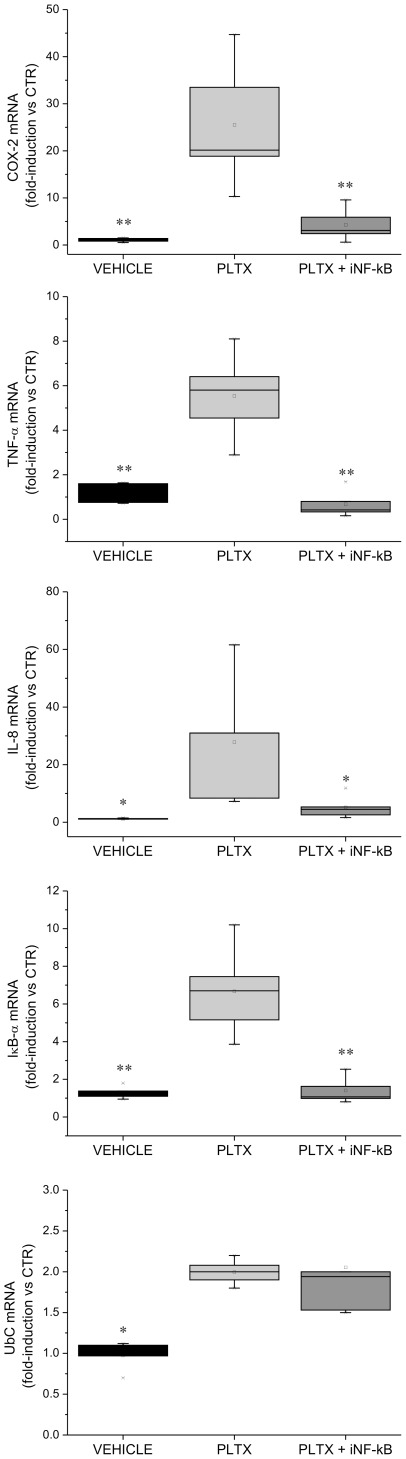
Inhibition of PLTX-induced COX-2, TNF-α, and IL-8 mRNA expression upon NF-κB inhibition. Total RNA was extracted from primary human macrophages pre-incubated 1 h with 50 µM andrographolide, an NF-κB inhibitor molecule (iNF-κB), and then exposed 4 h to PLTX (2 ng/ml). In parallel, macrophages were treated with PLTX or the vehicle alone. RNA was amplified with gene-specific primers. IκBα and UbC mRNA levels were used as positive and negative control, respectively. Expression data, normalized to the housekeeping B2M gene, were analysed by the 2^−ΔΔCT^ method and referred to the value obtained in untreated cells (CTR). The boxplots show the results of 5 independent experiments. Asterisks indicate statistical significance versus cells receiving PLTX (*p<0.05; **p<0.01).

Real-Time PCR analysis revealed that all the mRNAs analyzeds were significantly down-regulated in cells pre-treated with andrographolide as compared to cells left untreated and then exposed to palytoxin (Figure 7). Similar results were obtained with the OSTRTX extract (data not shown). IκB-α mRNA levels were monitored as a positive control and found to be strongly down-regulated as expected, based on the evidence that the IκB-α gene is under the direct transcriptional control of NF-κB (Figure 7). By contrast, UbC gene transcription, which is independent of NF-κB activation ([47] and unpublished results), was up-regulated up to 2-fold in toxin-treated cells as compared to the basal level (not shown), but its expression was unaffected by andrographolide, thus demonstrating specificity of action of the inhibitory molecule (Figure 7).

At the moment, little is known about intracellular signaling pathways modulated by PLTX and OSTRTX. Recent reports have indicated that palytoxin transmits signals through Mitogen-Activated Protein Kinases [28]. On the other hand, an important mechanism by which inflammatory genes are up-regulated in immune cells is through activation of MAPKs. Furthermore, MAPKs may participate in the signal transduction cascade leading to NF-κB activation [48], [49].

**Figure 8:**
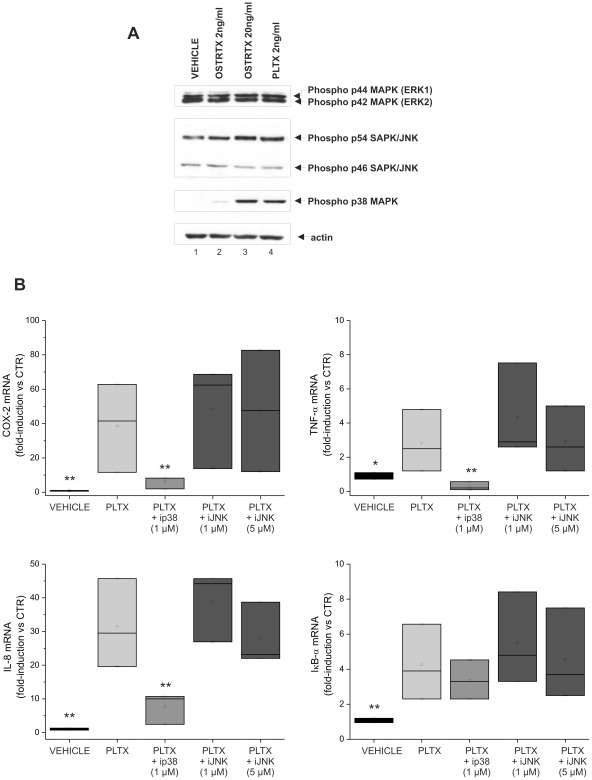
MAPKs activation and toxin-induced COX-2, TNF-α, IL-8 and IκBα mRNA expression upon p38 MAPK and JNK inhibition. (**A**) Western blot analysis of MAPK phosphorylation in macrophages incubated 4 h with the *O*. cf. *ovata* toxin extract (final OSTRTX concentration 2 ng/ml and 20 ng/ml) (lane 2, 3), 2 ng/ml PLTX (lane 4) or the vehicle (lane 1). 20 µg of whole cell extracts were separated by SDS-PAGE on 8% gel and submitted to western immunoblot with phospho-specific antibodies against ERK1/2, JNK and p38 MAPK. Actin was stained as a loading control. (**B**) Total RNA was extracted from macrophages pre-incubated 1 h with 1 µM p38 inhibitor (ip38) or 1 µM and 5 µM JNK inhibitor (iJNK), and then exposed 4 h to 2 ng/ml palytoxin. In parallel, macrophages were left untreated and then exposed to PLTX or to the vehicle alone. RNA was amplified with gene-specific primers. Expression data, normalized to the housekeeping B2M gene, were analyzed by the 2^−ΔΔCT^ method and referred to the value obtained in control cells (CTR). The boxplots show the results of 3 independent experiments, run in duplicate. Asterisks indicate statistical significance versus cells receiving only PLTX (*p<0.05; **p<0.01).

There is evidence that palytoxin-stimulated MAPK signalling can differ depending on the cell type [50]. On the other hand, most of the studies on PLTX toxicity have been performed using cell lines and, to the best of our knowledge, never in primary macrophages. Thus, based on these observations we wanted to preliminarily investigate the effects of PLTX and of the OSTRTX extract on MAPK activation in our cellular model. ERK activity was measured by immunoblot analysis and an antibody that specifically recognizes the dually phosphorylated active form of ERK1/2 (Figure 8A). The phospho-ERK immunoblot revealed that the kinases were already phosphorylated in whole cell extracts of vehicle-treated cells and the levels of the phosphorylated forms did not change upon cell exposure to PLTX or OSTRTX for 4 h (Figure 8A, compare lane 1 with 2–4). Depending on the cell donor, ERK phosphorylation appeared slightly down-regulated following toxin administration in some analyses (data not shown). A similar approach was used to test JNK and p38 MAPK activity. In both cases, kinases resulted activated by phosphorylation following incubation of the cells with PLTX or effective doses of OSTRTX (20 ng/ml) (Fig. 8A, lanes 2–4). At 2 ng/ml, OSTRTX only slightly affected the phosphorylation status of JNK p54 and p38 MAPK.

To elucidate the possible relationship between toxin-induced p38 MAPK and JNK activation and increased levels of inflammation-associated gene transcripts in macrophages, we used well known pharmacological blockers of kinase activity. Pre-treatment of macrophages with 1 µM SB 202190, a selective p38 inhibitor, reduced PLTX -induced COX-2, TNF-α and IL-8 mRNA levels by about 70–80% (Figure 8B). No effects were observed on IκB-α mRNA levels which directly relies on NF-κB activation (Figure 8B). By contrast, 1 µM JNK inhibitor failed to prevent or significantly decrease toxin-stimulated target gene mRNA accumulation. The inhibitor was also ineffective when the dose was raised to 5 µM (Figure 8B). Similar results were obtained with 20 ng/ml OSTRTX (data not shown). Collectively, inhibitor experiments indicate that p38 MAPK is involved, along with NF-κB, in the signalling cascade mediating synthesis and accumulation of transcripts encoding inflammation-related proteins.

## Discussion

PLTX is known as one of the most toxic non proteinaceaous compounds ever isolated. Depending on the exposure route, palytoxin and its *Ostreopsis* analogues have been postulated to cause several adverse effects on human health, including acute inflammatory reactions which seem more typical of cutaneous and inhalation contact (reviewed in [18]). In particular, subjects exposed to marine water in coincidence with *O.* cf. *ovata* blooms along the Italian, Spanish and French Mediteranean coasts, all reported a clinical picture where the most prominent and common symptoms were fever and irritative reactions of the upper and lower respiratory tracts, conjunctiva and skin, variously associated [18]. Significantly, when hospitalized, these patients benefited from a symptomatic treatment which also included corticosteroids or non-steroidal anti-inflammatory drugs [18]. On the whole, these evidences have led to hypothesize that PLTX and congener toxins may indeed exert a pro-inflammatory activity. Although several case reports ascribed to PLTX and ovatoxin exposure have been characterized from a clinical point of view, the presence of these toxins in the suspected causative specimen or targeted tissue has not always been confirmed [18]. In addition, since the source of contamination *in vivo* is obviously represented by complex biological matrices, at present it cannot be excluded that cell fragments and/or other chemical substances, other than palytoxin and/or ovatoxins themselves, could be responsible for the observed effects. Thus, this study was undertaken with the aim to establish whether PLTX and, possibly, its congener toxins from *Ostreopsis* cf. *ovata*, a species known to cause poisoning in the Mediterranean area, may activate pro-inflammatory signalling pathways in cells of the immune system. To this end, we have tested commercially available purified PLTX and compared its activity to that of a semi-purified toxin extract from *O.* cf. *ovata* cells containing, other than 1.2% putative PLTX, all the ovatoxins so far known (ovatoxin-a, -b, -c, -d, -e) and a new one, named ovatoxin-f (Table 1). Among the various *O.* cf. *ovata* extracts so far characterized [11], [35], [36], the toxin extract obtained from the CBA2-122 clone was selected as it presented the most complete ovatoxin profile, in that it contains all the ovatoxins so far known. To the best of our knowledge, this is the first investigation which attempts to gain information on the biological activity of ovatoxin.

Our data clearly demonstrate that in primary human macrophages, which represent the first line of defence at contact sites, PLTX and the *O.* cf. *ovata* semi-purified toxin extract induce a significant accumulation of gene transcripts, the products of which are involved in inflammation (Figure 2). Selected targets consisted of COX-2, TNF-α and IL-8. Cycloxigenase-2 catalyses the conversion of arachidonic acid to prostaglandins, resulting in pain and inflammation [51]. It has been previously demonstrated that pM concentrations of palytoxin stimulate production of prostaglandins and that this event requires new protein synthesis [27], [52]. However, these studies did not explore the nature of the signalling pathway leading from palytoxin binding to arachidonic acid metabolism. Thus, our evidence adds a new piece to the puzzle, suggesting that PLTX-stimulated prostaglandin production may occur as the consequence of PLTX-induced NF-κB and p38 MAPK activation which leads to increased levels of COX-2 mRNA.

Similarly to COX-2 mRNA, the other targets analysed (i.e. TNF-α and IL-8) resulted significantly up-regulated by PLTX as compared to vehicle-treated cells (Figure 2). The accumulation of transcripts encoding TNF-α and IL-8 in macrophages in response to PLTX is in agreement with induction of an acute inflammatory reaction. Indeed, TNF-α is a pro-inflammatory cytokine and mediator of many immune functions which is produced during acute phase reaction, particularly by activated macrophages [53]. IL-8 is a potent pro-inflammatory chemokine that can promote rapid migration of neutrophils to sites of infection and inflammation [54]. Compared to PLTX, the OSTRTX extract, although at a 10-fold higher toxin concentration, produced the same effect at the molecular level (Figure 2), suggesting that ovatoxins may exert a PLTX-like activity. Unfortunately, because the *O.* cf *ovata* toxin extract was partially purified and it also contains a putative PLTX, a direct correlation between ovatoxins and increased mRNA levels of inflammation-related genes cannot be established at the moment. However, on one hand, the very good overlap between the effects observed in response to PLTX and to the O. cf. ovata toxin extract and the similarity in the chemical structure between ovatoxins and PLTX would exclude the involvement of other contaminant substances which may be present in the extract. On the other hand, the very high relative abundance of ovatoxins (99% of OSTRTX) strongly suggests that OVTXs, rather than the putative PLTX, may directly induce or at least contribute significantly to the activity displayed by the OSTRTX extract. In addition, since toxicity may be either positively or negatively affected by impurities, at the moment it is difficult to establish whether ovatoxins may be less powerful than palytoxin. Similarly, it cannot be determined whether all the ovatoxin types or only some of them have biological activity. All these aspects will be elucidated only once it becomes possible to purify single components in sufficient amounts to be tested in *in vitro* cellular systems.

Once established that PLTX and the OSTRTX extract could indeed promote the synthesis/accumulation of mRNAs encoding inflammation-related proteins, we next investigated signalling cascades possibly involved. This aspect was approached by testing the involvement of new toxin molecular targets which mediate immune/inflammatory reactions, such as transcription factor –κB, and by reconsidering, in the context of inflammation, the role of signalling mediators already known to be modulated by PLTX (i.e. MAPKs). NF-κB resulted activated in cells exposed to PLTX and to the OSTRTX extract via the canonical pathway which involves reduction of IκB-α protein levels (Figure 5A) and subsequent NF-κB nuclear translocation (Figure 6). When compared to PLTX, the OSTRTX extract displayed totally overlapping effects on the NF-κB pathway only at the OSTRTX dose that was effective in accumulating inflammation-associated mRNAs in macrophages (i.e. 20 ng/ml). Thus, although a direct relationship between OSTRTX, particularly ovatoxins, and NF-κB activation cannot be established, for the reasons explained above, these results strongly suggest that these toxins may indeed engage the same signalling pathways activated by PLTX. On the other hand, when cells were pre-treated with an NF-κB inhibitor, which prevents NF-κB DNA binding, the levels of all the target mRNAs, including the IκB-α mRNA which is directly synthesized following NF-κB activation, resulted down-regulated by 70–80% as compared to cells receiving only PLTX or the OSTRTX extract (Figure 7). This result provides the proof that NF-κB activation is indeed functionally involved in the toxin-induced pro-inflammatory signalling cascade leading to the accumulation of inflammation-related transcripts. By contrast, no effect was observed on toxin-induced transcriptional activation of the UbC gene. Ubiquitin C is one of the four functional genes which encode ubiquitin (Ub) in mammalian cells. The UbC gene is typically referred to as a stress-inducible gene and a great bulk of literature describes the transcriptional induction of ubiquitin upon cell challenge with different types of stress [55]. The finding that ubiquitin is up-regulated in PLTX- and OSTRTX-treated cells is in agreement with previous observations in literature which demonstrate that the cytotoxic pathway triggered by PLTX involves changes in the cellular pool of some stress response proteins [56]. Due to the pivotal role played by ubiquitin in many cellular processes, including protein degradation of stress-damaged proteins, this observation undoubtedly deserves further investigations. For example, the Na+/K+ ATPase, which is the main target of PLTX, has been shown to be ubiquitinated and subsequently degraded within lysosomes under both basal and stress conditions [57]. Thus, it could be hypothesized that induction of ubiquitin may favor removal of toxin-“damaged” Na+/K+ ATPase, as a mechanism of cellular defence against PLTX toxicity. On the other hand, it should also be considered that very preliminary evidence, reported in this paper, indicates that palytoxin treatment may neutralize macrophage lysosomal protease activity in intact cells through a mechanism that is still to be defined and that could contribute to PLTX toxicity (Figure 5B, C). On the whole, these observations suggest that the two main cellular proteolytic systems (i.e. the ubiquitin/proteasome and the lysosomal pathways) could have a role in the toxic action of these marine biotoxins.

It has been well established that mitogen-activated protein kinases can mediate palytoxin-stimulated signalling [28]. In our experimental model, both p38 MAPK and JNK were indeed phosphorylated upon exposure of macrophages to PLTX or to the OSTRTX extract (Figure 8A). In the latter case differences with respect to vehicle-treated cells were already detectable at the lower concentration tested, although a clear accumulation of the phospho-activated forms were observed only at the highest OSTRTX dose (i.e. 20 ng/ml). Studies performed with chemical inhibitors demonstrate that inhibition of p38, but not JNK, strongly suppresses toxin-induced accumulation of inflammation-related transcripts, thus suggesting a role for p38 in the pro-inflammatory signalling activity of PLTX and OSTRTX/ovatoxins (Figure 8B). Strong down-regulation of TNF-α, IL-8 and COX-2 mRNA expression in monocyte-derived macrophages upon inhibition of p38 MAPK has been reported by others [58]. Indeed, in inflammatory processes, the p38 MAPK signal transduction route regulates production and expression of cytokines and other inflammatory mediators. In particular, MAPKs have been widely reported to regulate gene expression through the direct phosporylation and activation of transcription factors, which are responsible for expression of target genes [59]. However, post-transcriptional regulation of inflammatory gene expression has also been linked with the p38 pathway [60]. While p38 MAPK, but not JNK, seems to participate in the toxin-induced pro-inflammatory signalling cascade, at the moment our data seem to exclude a role for ERK. Indeed, ERK1/2 were constitutively phosphorylated in macrophages, in accordance with the knowledge that they are activated by the process of adherence [38]. Significantly, their phosphorylation state was unaffected by toxin treatment, an observation which would exclude a role in toxin-induced pro-inflammatory signalling. However, it has been recently demonstrated that palytoxin also activates another MAPK family member, called ERK5 [61]. Like other MAPKs, ERK5 is activated by variety of stimuli, including growth factors, G-protein-coupled receptor (GPCR) agonists, cytokines, and stress [62]. This kinase has not been studied as extensively as the other members of the MAPK family, but there is very preliminary evidence which suggests a possible role in promoting inflammation [63], [64]. Thus, further analyses will be necessary to establish whether ERK5 participates in toxin-induced macrophage activation.

In conclusion, data reported in this paper demonstrate for the very first time that palytoxin and, most likely, its congener *Ostreopsis* cf. *ovata* toxins, induce a significant increase in the levels of mRNAs encoding inflammation-related proteins in cells of the immune system, an observation which suggest that these toxins have the potential to exert a pro-inflammatory activity. At the cellular level, the activity of these biotoxins is sustained by their ability to activate both the NF-κB and the p38 MAPK signalling pathways, although upstream molecular targets remains to be determined and downstream effectors to be detected. On one hand, the evidence concerning the involvement of NF-κB and p38 MAPK justifies the beneficial effects of non-steroidal anti-inflammatory drugs (NSAIDs) and corticosteroids in patients exposed to these toxins. Indeed, it is known that these molecules are able to interfere with these pathways [65]–??[67]. On the other hand, the identification of specific molecular targets of palytoxin and its analogues, besides contributing to expand the still limited knowledge of the intracellular signalling cascades affected by these toxins, may have important implications in setting up more rational and focused pharmacological interventions, replacing currently used symptomatic treatments.

## Materials and Methods

### Batch culture of *O.* cf. *ovata*

The *Ostreopsis* cf. *ovata* CBA2-122 used in this study was originally isolated from a field sample collected at Ancona, north-western Adriatic Sea – Italy, during the autumn 2008. Cultures were grown in 1 litre glass flasks containing 600 ml sterilized f/4 medium with an initial cell amount of 3.0×10^4^ cells [68]. The temperature was set at 23±1°C. Light was provided by cool white fluorescent bulbs (photon flux of 100 µE m^−2^ s^−1^) on a standard 14 h light-10 h dark cycle. For cells enumeration, culture samples were fixed with Lugol's iodine and counted using the Utermhöl method [69]. Cultures were harvested by centrifugation at 4,000× g for 15 min at room temperature, and pellets were pooled in one single sample containing approximately 3.0×10^6^ cells. Cell pellet was stored at −80°C until toxin extraction.

### *O.* cf. *ovata* toxin extraction and clean-up

*O.* cf. *ovata* cell pellet was extracted with 60 ml of methanol/water (1∶1 v/v) 0.2% acetic acid following the procedure previously reported [11]. An aliquot corresponding to 75% of the crude extract was dried under vacuum, the residue was re-dissolved in 0.5 ml of acetonitrile/water (1∶1 v/v), and subjected to purification by semi-preparative HR LC-MS.

### HR LC-MS experiments

HR LC-MS analyses were performed on an Agilent 1100 LC binary system (Palo Alto, CA, USA) coupled to a hybrid linear ion trap LTQ Orbitrap XL™ Fourier transform mass spectrometer (FTMS) equipped with an ESI ION MAX™ source (Thermo-Fisher, San Josè, CA, USA). HR full MS experiments (positive ions) were acquired in the range *m/z* 800–1400 at resolution setting 30.000. The following source settings were used: spray voltage = 4 kV, capillary temperature = 290°C, capillary voltage = 45 V, sheath gas = 35 and auxiliary gas = 1 (arbitrary units), tube lens voltage = 165 V (*m/z* 800–1400) or 250 V (*m/z* 2000–3000). Purification of the *O.* cf. *ovata* crude extract was accomplished on a 10 mm Gemini C18 (10×250 mm i.d.) column maintained at room temperature and eluted at 2 ml/min with water (eluent A) and 95% acetonitrile/water (eluent B), both containing 30 mM acetic acid. A gradient elution (20–100% B over 40 min) and a sample injection volume of 80 ml were used. This procedure led to obtain a semi-purified extract containing putative palytoxin and ovatoxins (OVTX-a, -b, -c, -d + -e and –f).

Toxins contained in the semi-purified extract were quantified by using LC conditions reported previously [11]. Extracted ion chromatograms (XIC) were obtained from the HR full MS spectra by selecting the most abundant peaks of the [M+2H-H_2_O]^2+^ and [M+H+Ca]^3+^ ion clusters of each compound (Table 1). A mass tolerance of 5 ppm was used. Peak areas were measured and interpolated within the calibration curve of palytoxin standard (Wako Chemicals GmbH, Neuss, Germany) at five levels of concentrations (50, 25, 12.5, 6.25, and 3.13 ng/ml). Linearity of the calibration curve was indicated by a correlation coefficient (R^2^) of 0.9980. In lack of standards for ovatoxins and on the base of structural similarities between palytoxin and ovatoxins, their molar responses were assumed to be the same as that of palytoxin.

### Endotoxin content determination

Endotoxin content of palytoxin stock solutions and of the OSTRTX extract was determined by a kinetic Limulus amoebocyte lysate (LAL) assay using an Endochrome-K assay kit (Charles River Laboratories International Inc., Wilmington, MA, USA), according to the manufacturer's instructions. Samples were serially diluted from 1∶50 (v/v) to 1∶5000 (v/v) in LAL reagent water and assayed in triplicate. Absorbance at 405 nm was monitored for 30 min at 2 min intervals, starting immediately after LAL reagent addition. At the end of the monitoring interval, endotoxin activity was calculated from a control standard endotoxin curve. Possible interference of PLTX and of the OSTRTX extract with the LAL enzymatic cascade was excluded by an inhibition/enhancement test run in parallel.

### Monocyte-derived human macrophages preparation and treatment

Mononuclear cells (MCs) were isolated from Pathogen-negative buffy coats, obtained from the Blood Transfusion Center of the Hospital “S. Maria della Misericordia” – Urbino (Italy), by separation on Lymphoprep (Axis Shield, Oslo, Norway; specific density 1.077). Buffy coats were prepared from the blood of adult volunteers who signed an informed consent form before donation and samples were provided as anonymous. The use of primary human blood cells in the context of the present study has been approved by the research ethics committee of the University of Urbino. MCs (12×10^6^ cells/well) were seeded onto 6-well plastic culture plates (Greiner Bio-One GmbH, Frickenhausen, Germany) and monocytes were separated from lymphocytes by adherence overnight at 37°C as previously described [70]. After removal of non-adhering cells by repeated washes, cells were cultured in RPMI 1640 medium supplemented with 10% fetal bovine serum (heat inactivated for 30 min at 56°C), 2 mM glutamine, 100 µg/ml streptomycin, and 100 U/ml penicillin (all purchased from Cambrex Bioscience, Verviers, Belgium), at 37°C in a 5% CO_2_ atmosphere. The culture medium was changed every 2 days and after 7 days of culture the vast majority of the adherent cells were differentiated macrophages, as revealed by immunostaining with an anti CD14 antibody (R&D Systems Inc., Minneapolis, MN, USA). On the 8^th^–10^th^ day of culture, monocyte-derived macrophages were incubated with 2 ng/ml palytoxin (Wako Chemicals GmbH, Neuss, Germany), or with the *O.* cf. *ovata* semi-purified toxin extract to a final OSTRTX concentration in cell medium of 2 ng/ml and 20 ng/ml. Untreated and vehicle-treated (i.e. 0.05%. Methanol) cells were used as controls. In some experiments macrophages were pre-treated 1 h with the following chemicals before toxin administration: 1 µM SB202190 (p38 inhibitor), 1 µM and 5 µM SP 600125 (JNK inhibitor) and 50 µM andrographolide (NF-κB inhibitor), all obtained from Sigma Aldrich (Steinheim, Germany).

### RNA isolation and quantitative Real-Time PCR

Total RNA was isolated using the RNeasy (plus) mini kit (Qiagen Inc. Valencia, CA, USA) and its concentration accurately determined using the Nanodrop ND-1000 System (NanoDrop Technologies, Wilmington, DE, USA). First-strand cDNA was synthesized with the SuperScript First-Strand Synthesis System for RT-PCR (Invitrogen, Eugene, OR, USA) and oligo-dT primers (0.5 µg/µl) in a final volume of 20 µl, according to the manufacturer's instructions. The synthesized cDNAs were used as templates in SYBR green quantitative Real-Time PCR (qRT-PCR) assays, performed with the Hot-Rescue Real-Time PCR kit (Diatheva s.r.l., Fano, Italy). PCR reactions were set up in a volume of 25 µl containing 1× Hot-Rescue Real-Time Master Mix, 0.2 µM of gene specific primers, 0.625 units of Hot-Rescue DNA polymerase, 5 µl (0.6 ng/µl) of the RNase H-treated cDNA stock and the MgCl_2_ concentration specified below for the various targets investigated. DNA amplifications were carried out in 96-well reaction plates using ABI PRISM 7700 Sequence Detection System platform (Applied Biosystems, Foster City, CA, USA). qRT-PCR primers (obtained from Sigma-Genosys Ltd, Haverhill, UK) were designed using Primer Express version 2.0 and tested to confirm the appropriate product size and optimal concentrations. Primer sequences, as well as the relative MgCl_2_ concentration used were: COX-2 forward, 5′-CACCCATGTCAAAACCGAGG-3′ and reverse, 5′-CCGGTGTTGAGCAGTTTTCTC-3′ (3.5 mM MgCl_2_); IL-8 forward, 5′-ATGACTTCCAAGCTGGCCGT-3′ and reverse, 5′-CAGCCCTCTTCAAAAACTTCTCC-3′ (2.5 mM MgCl_2_); TNF-α forward, 5′- GCCCAGGCAGTCAGATCATCTTC -3′ and reverse, 5′-TGCCCCTCAGCTTGAGGGT-3′ (2.5 mM MgCl_2_); IκB-α forward, 5′- CGCACCTCCACTCCATCCT-3′ and reverse, 5′- ACATCCAGCCCCACACTTCAAC -3′ (3.5 mM MgCl_2_); UbC forward, 5′-GTGTCTAAGTTTCCCCTTTTAAGG-3′ and reverse 5′-TTGGGAATGCAACAACTTTATTG-3′ (5 mM MgCl_2_); β2-microglobulin (B2M) forward, 5′-GCCTGCCGTGTGAACCAT-3′ and reverse, 5′-CATCTTCAAACCTCCATGATGCT-3′ (3.5 mM MgCl_2_). Cycle conditions were 95°C for 10 min followed by 40 cycles of 15 s at 95°C, 15 s at 60°C and 30 s at 72°C. Amplification plots were analyzed using SDS 1.9.1 software (Applied Biosystems) and relative expression data were calculated with the 2^−ΔΔCT^ method [71]. Thus, the relative abundance of the various genes investigated, normalized to the housekeeping B2M gene, was expressed as percent amount in cells receiving PLTX, different concentrations of OSTRTX or the vehicle alone with respect to the reference sample, represented by untreated cells.

### Optical microscopy

Macrophages exposed 4 h to 2 ng/ml PLTX and 20 ng/ml OSTRTX were observed with an Olympus IX51 microscope (Olympus Corporation, Tokio, Japan). Vehicle-treated cells were used as control. Pictures were taken at a magnification of 40× directly in culture medium without cell fixation in order to avoid artifacts.

### Cell toxicity assay

Cytotoxicity was assessed by using a CellTiter-96 aqueous one solution kit from Promega (Madison, WI, USA). This assay is based on the reduction of the MTS reagent [3-(4,5-dimethylthiazol-2yl)-5-(3-carboxymethoxyphenyl)-2-(4-sulfophenyl)2H-tetrazolium, inner salt] into a colored formazan product that is soluble in tissue culture medium. This conversion is accomplished by NADPH or NADH produced by dehydrogenase enzymes in metabolically active cells. The quantity of formazan product, as measured by the absorbance at 490 nm, is directly proportional to the number of living cells in culture.

### Preparation of whole-cell lysates

For whole-cell extract preparation, macrophages were directly harvested in SDS buffer: 50 mM Tris-HCl, pH 7.8, 0.25 M sucrose, 2% (w/v) SDS, supplemented with a commercially available cocktail of protease (Roche Applied Science, Indianapolis, IN, USA) and phosphatase (1 mM NaF, 1 mM Na_3_VO_4_) inhibitors. Lysates were boiled for 5 min, then sonicated at 100 Watts for 20 sec. Cell debris was removed by brief centrifugation (10 min at 12,000× g). Protein content was determined by the Lowry assay [72].

### Nuclear-cytoplasmic subcellular fractionation

Cytosolic and nuclear extracts were obtained by low salt/detergent cell lysis followed by high salt extraction of nuclei as previously described [73]. After treatment, cells were extensively washed with cold PBS and lysed with Buffer A [10 mM Hepes/KOH pH 7.9, 1.5 mM MgCl_2_, 10 mM KCl, 1 mM dithiothreitol (DTT), 0.2 mM EDTA, 0.1% Nonidet-P40, supplemented with a cocktail of protease (Roche Applied Science) and phosphatase inhibitors]. To completely block *in vitro* degradation processes the buffer was further supplemented with the following protease inhibitors (Buffer A PLUS): 25 µg/ml leupeptin (Sigma Aldrich), 10 µg/ml pepstatin (Sigma Aldrich), 4 mM AEBSF (Roche Applied Science), 100 µM MG-132 (Enzo Life Sciences Inc., NY, USA). The cell suspension was then chilled on ice for 10 min before centrifugation at 10,000× g. The supernatant, corresponding to the cytosolic fraction, was then transferred to a fresh tube, while the resultant pellet was suspended in Buffer B [20 mM Hepes/KOH pH 7.9, 25% glycerol, 0.42 M NaCl, 1.5 mM MgCl_2_, 1 mM DTT, 0.2 mM EDTA, supplemented with the cocktail of protease (Roche Applied Science) and phosphatase inhibitors, 25 µg/ml leupeptin, 10 µg/ml pepstatin, 4 mM AEBSF and 100 µM MG-132] and incubated on ice for 20 min before being centrifuged at 10,000× g. Nuclear extract supernatant was collected, diluted 1∶4 in Buffer C [20 mM Hepes/KOH pH 7.9, 20% glycerol, 50 mM KCl, 1 mM DTT, 0.2 mM EDTA, 4 mM AEBSF, 25 µg/ml leupeptin, 5 µg/ml pepstatin, 100 µM MG-132] and stored in aliquots at −80°C until use. The resulting pellet, containing nuclear membranes and cytoskeleton, was lysed directly in SDS PAGE sample buffer.

### Western immunoblotting

Protein extracts were resolved by SDS-PAGE and gels were electroblotted onto a nitrocellulose membrane (0.2 µm pore size) (BioRad laboratories Inc., Milano, Italy). The blots were probed with the primary antibodies listed below and bands were detected using horseradish peroxidase-conjugated secondary antibody (BioRad Laboratories Inc.). Peroxidase activity was detected with the enhanced chemiluminescence detection method (ECL Kit, Amersham Biosciences, Arlington Heights, IL, USA). The antibodies used in this study were: anti-p65 (C-20, sc-372) and anti-IκBα (C-21, sc-371) from Santa Cruz Biotechnology Inc. (Santa Cruz, CA, USA); anti-actin (A 2066) from Sigma-Aldrich; anti-Phospho-p44/42 MAPK (Erk1/2) (Thr202/Tyr204), anti Phospho-SAPK/JNK (Thr183/Tyr185) and anti Phospho-p38 MAPK (Thr180/Tyr182) from Cell Signaling Technology (Beverly, MA, USA).

### Electrophoretic Mobility Shift Assay (EMSA)

Upper strand (5′-TCAACAGAGGGGACTTTCCGAGAGGCC-3′) and reverse-complement phosphodiester oligonucleotides, containing the NF-κB binding sequence (underlined), found in the enhancer of the immunoglobulin light chain gene (Igκ) were custom synthesized by Thermo Fisher Scientific GmbH (Ulm, Germany) as HPLC-purified products. As control, a double-stranded ODN, containing the consensus sequence of the unrelated transcription factor YY1 (5′-CGCTCCGCGCCATCTTGGCGGCTGGT-3′) was used as competitor. The Igκ double-stranded ODN was 5′ end-labeled with [γ-^32^P] ATP (Perkin Elmer Inc., Waltham, MA, USA) and T4 polynucleotide kinase (T4 PNK, Roche Applied Science). Nuclear extracts (5 µg) were preincubated with 3 µg of double-stranded non-specific DNA competitor poly(dI-dC) (Amersham Biosciences) for 10 min on ice in binding buffer (20 mM Hepes-KOH, pH 7.9, 0.1 M KCl, 5% (v/v) glycerol, 0.2 mM EGTA, 0.2 mM EDTA, 1 mM DTT). After this time, a ^32^P-end-labeled DNA probe was added to the mixtures at a final concentration of 4.4 nM and the incubation was continued for an additional 30 min. Reaction mixtures were then subjected to electrophoretic separation on 5% native polyacrylamide gels (29∶1 cross-linked) in Tris-glycine buffer (25 mM Tris base, 192 mM glycine). DNA/protein complexes were detected by exposing the dried gel in a Molecular Imager (BioRad Laboratories Inc.). For competition experiments, nuclear extracts were incubated with a 50-fold excess of double-stranded competitor ODN for 10 min before adding the ^32^P-labeled probe. For supershift experiments, nuclear extracts were incubated with 2 µg of anti p50 (Cell Signaling Technology), anti-p65 (C-20, sc-372X) or anti YY1 (C-20, SC-281X) antibody (Santa Cruz Biotechnology Inc.). Detection of NF-κB/DNA complex formation was performed in a GS-250 Molecular Imager (BioRad Laboratories Inc.).

### Statistical analysis

Statistical analysis of data was performed by ANOVA for repeated measurement, followed by the Tukey-Kramer multiple comparison test (for n groups >2) or with the paired t-test (for n groups = 2), using GraphPad InStat version 3.0.6 for Windows (GraphPad Software). Differences between values were assumed statistically significant at p<0.05 (*) and very significant at p<0.01 (**).
